# Risk factors for progression of chronic kidney disease in the EPPIC trials and the effect of AST-120

**DOI:** 10.1007/s10157-017-1447-0

**Published:** 2017-07-24

**Authors:** Gerald Schulman, Tomas Berl, Gerald J. Beck, Giuseppe Remuzzi, Eberhard Ritz, Miho Shimizu, Mami Kikuchi, Yuko Shobu

**Affiliations:** 1 Vanderbilt University School of Medicine, Nashville, TN USA; 2 University of Colorado Health Sciences Center, Denver, CO USA; 3 Cleveland Clinic Foundation, Cleveland, OH USA; 4 Unit of Nephrology and Dialysis, Azienda Ospedaliera Papa Giovanni XXIII, Bergamo, Italy; 5 IRCCS Istituto di Ricerche Farmacologiche Mario Negri, Bergamo, Italy; 6 Department of Biomedical and Clinical Sciences, University of Milan, Milan, Italy; 7 University of Heidelberg, Heidelberg, Germany; 8 Mitsubishi Tanabe Pharma Corporation, Tokyo, Japan; 9 Kureha Corporation, 3-26-2, Hyakunin-cho, Shinjuku-ku, Tokyo, 169-8503 Japan

**Keywords:** AST-120, CKD, CKD progression, Clinical trial, Spherical carbon adsorbent, Uremic toxins

## Abstract

**Background:**

Two randomized, double-blind, placebo-controlled trials (EPPIC-1 and EPPIC-2) investigated the efficacy and safety of AST-120, an oral spherical carbon adsorbent, in adults with chronic kidney disease (CKD). While the benefit of adding AST-120 to standard therapy was not supported by these trials, we performed a post hoc analysis to focus on CKD progression and to determine the risk factors for the primary endpoint in the EPPIC trial population.

**Methods:**

In the EPPIC trials, patients were randomly assigned 1:1 to treatment with AST-120 or placebo. The primary endpoint was a composite of dialysis initiation, kidney transplantation, or doubling of serum creatinine. The EPPIC trial pooled population was evaluated with the same statistical methods used for analysis of the primary and secondary efficacy endpoints. The trials were registered on ClinicalTrials.gov (NCT00500682 [EPPIC-1] and NCT00501046 [EPPIC-2]).

**Results:**

An analysis of the placebo population suggested baseline urinary protein to urinary creatinine ratio (UP/UCr) ≥1.0 and hematuria were independent risk factors for event occurrence and eGFR lowering. Analysis of the high risk patients revealed a difference in the primary endpoint occurrence between treatment groups, if angiotensin-converting enzyme inhibitors and/or angiotensin receptor blockers were administered (hazard ratio 0.74, 95% confidence interval 0.56–0.96). Also, the eGFR changes from baseline in the AST-120 group were smaller than that in the placebo group (*P* = 0.035).

**Conclusions:**

CKD progression may have an association with baseline UP/UCr and hematuria. Treatment with AST-120 may delay the time to the primary endpoint in patients with progressive CKD receiving standard therapy, thus warranting further investigation.

**Electronic supplementary material:**

The online version of this article (doi:10.1007/s10157-017-1447-0) contains supplementary material, which is available to authorized users.

## Introduction

The global increase in the incidence of end stage renal disease (ESRD) is a major issue in health economics presently [1]. Chronic kidney disease (CKD) has been documented as a risk factor for cardiovascular disease [2], and it is widely accepted that CKD can affect a patient’s prognosis in addition to affecting health economics.

Recently, it has been established that the prognosis of patients with CKD differs depending on the underlying diseases and the presence of certain factors, highlighting the importance of understanding the progression of CKD. Coresh et al. reported that in their cohort study, ESRD and mortality risks could be predicted by measuring estimated glomerular filtration rate (eGFR) decline over 2 years [3].

AST-120 (Kureha Corporation, Tokyo, Japan) (also known as Kremezin^®^), an oral spherical carbon adsorbent, was approved in 1991 in Japan for delaying the initiation of dialysis and ameliorating symptoms of uremia in patients with progressive CKD and has been a component of multimodal therapies for CKD [4]. AST-120 has also been approved for use in Korea (2005), Taiwan (2008), and the Philippines (2010). AST-120 reduces the concentrations of indoxyl sulfate (IS), a uremic toxin, which enhances the progression of CKD and may be related to cardiovascular disease, in the systemic circulation. AST-120 lowers IS levels by preventing the absorption of indole, a tryptophan breakdown product and a precursor of IS, from the gastrointestinal tract, which is the presumed mechanism underlying AST-120’s effect of slowing the progression of CKD [5].

Two large, multinational, randomized, placebo-controlled, phase 3 trials [Evaluating Prevention of Progression In Chronic kidney disease (EPPIC-1 and EPPIC-2)] evaluated the efficacy of adding AST-120 to standard therapy in adults with CKD [6]. In both trials, the primary endpoint was a triple composite of time from randomization to renal disease progression, as indicated by initiation of dialysis, kidney transplantation, or doubling of serum creatinine (sCr). The primary analysis of the intent-to-treat (ITT) population for each EPPIC trial showed the percentage of patients reaching the primary endpoint was similar in the AST-120 and the placebo groups [EPPIC-1 35.6 vs 35.3%, respectively; hazard ratio (HR) 1.03, 95% CI 0.84–1.27, *P* = 0.78] (EPPIC-2 34.4 vs 36.8%, respectively; HR 0.91, 95% CI 0.74–1.12, *P* = 0.37).

The failure to detect a significant difference between the treatment groups in the EPPIC trials may have been due to a longer than expected time to renal disease progression among the placebo-treated patients. The EPPIC pooled placebo analysis revealed that higher urinary protein to urinary creatinine ratio (UP/UCr) and the prevalence of hematuria were key factors for predicting a more rapid eGFR decline [6]. In the present analysis, we used the pooled EPPIC trial data to determine risk factors for both primary endpoint occurrence and depression of renal function, and then investigated if AST-120 had an effect in the sub-populations with those risk factors.

## Materials and methods

Details of the EPPIC trials including the patient flow diagram and full materials and methods for the EPPIC trials are published in the Supplemental Materials and Methods for Ref. [6] and Supplement 1.

### Study design

The EPPIC trials were conducted between July 2007 and February 2012 at 239 international sites in 13 countries to compare the effects of AST-120 with those of placebo with regard to renal outcomes in patients with moderate-to-severe CKD receiving standard therapy. Patients were randomly assigned 1:1 to receive treatment with 9 g/day AST-120 or placebo. AST-120 (administered as ten 300 mg capsules three times daily) or placebo was administered with meals and at least 1 h after concomitant medication. Phosphate binders could be administered simultaneously with the study medication.

### Patients

Eligible patients were aged 18 years or older with moderate-to-severe CKD [defined as sCr level of 2.0–5.0 mg/dL (in male patients) or 1.5–5.0 mg/dL (in female patients) at screening] who were not expected to require dialysis or renal transplantation within 6 months of trial entry and who were expected to survive for 1 year or greater. All patients were required to demonstrate proteinuria or progressive deterioration in renal function based on either UP/UCr ≥0.5 at screening or an increase in sCr level by >10% at the second evaluation conducted 3 months after the screening. If a patient was receiving antihypertensive therapy, treatment must have been stable and must have included either angiotensin-converting enzyme inhibitor/angiotensin receptor blocker (ACEI/ARB) unless contraindicated.

### Outcome

The primary endpoint for this analysis was occurrence of any component of the triple composite endpoint: initiation of dialysis, kidney transplantation, or doubling of sCr levels. ESRD was defined as initiation of dialysis or kidney transplantation.

One of the secondary endpoints, eGFR, was evaluated in this report. The eGFR was calculated using abbreviated MDRD GFR equation [7]. Change from baseline in eGFR (%) was calculated during the first 96 weeks of treatment to elucidate the degree of renal disease progression.

### Statistical analysis

A pooled population of both EPPIC trials was used for all analyses.

The univariable and multivariable analyses were applied using the demographic and the baseline clinical characteristic as covariates to find the risk factors for the primary and the secondary endpoints. The Cox proportional hazards regression model and the mixed-effects model were applied for the primary and the secondary endpoints, respectively.

For post hoc subgroup analyses, the same statistical methods used for analysis of the primary efficacy endpoint in the EPPIC trials were applied. The primary composite endpoint was analyzed using stratified Cox proportional hazards regression model; covariate adjustments were CKD etiology, baseline sCr level, and region.

The Kaplan–Meier (K–M) method and stratified Cox regression analysis were applied to compare time to the onset of primary endpoint between AST-120 and placebo groups. The secondary endpoint, change from baseline in eGFR (%), was analyzed using the mixed-effects model for repeated measures and analysis of covariance (ANCOVA).

The effect of AST-120 in the subgroups with factors predicting rapid disease progression was then assessed. Hematuria status was classified as negative, “trace”, +1, +2, and +3, and patients with “trace” levels were included in the hematuria-positive population in this analysis.

## Results

**Fig. 1:**
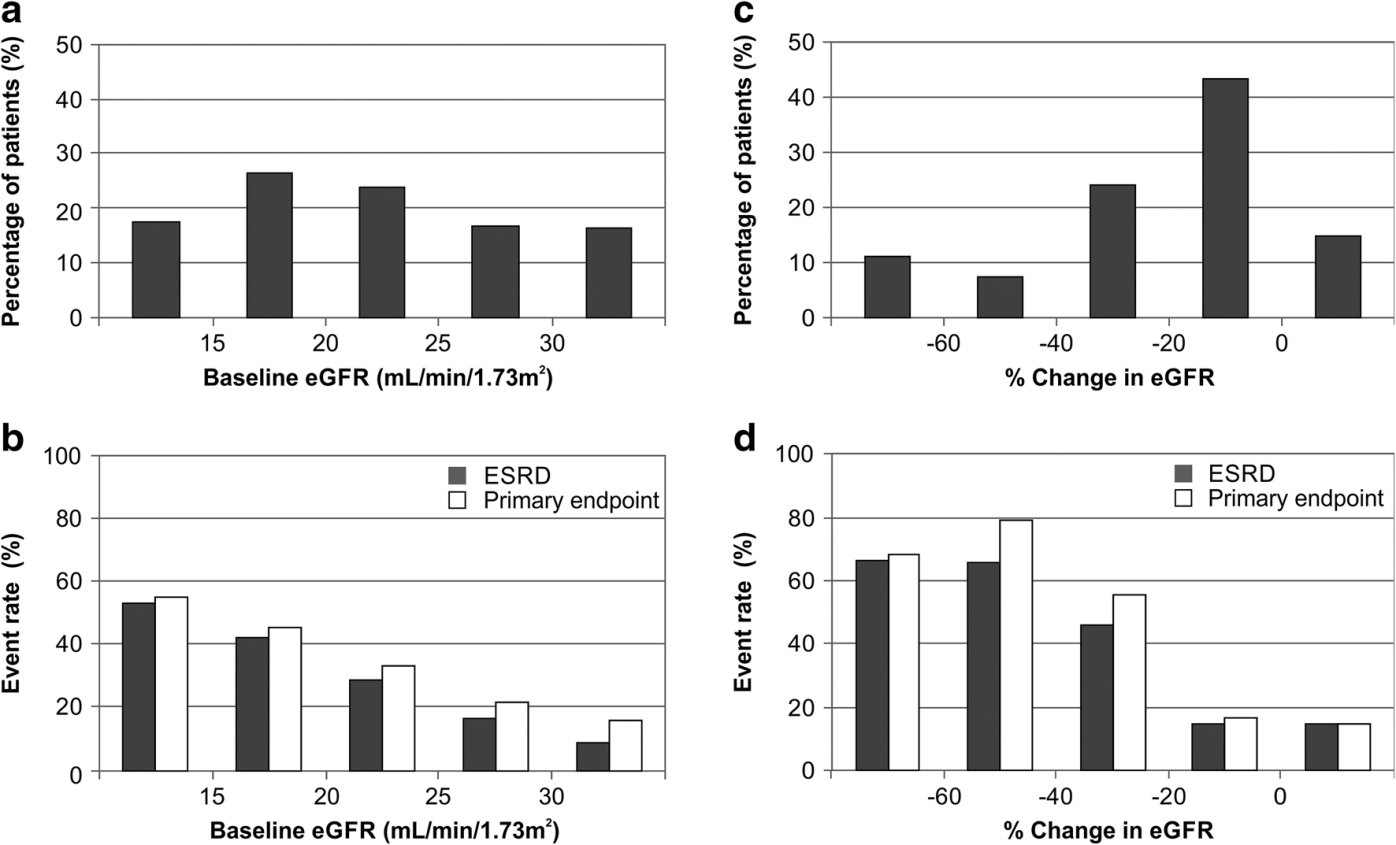
Distribution of baseline eGFR and eGFR change populations (pooled placebo ITT population). **a** Percentage of patients in terms of baseline eGFR, **b** event rates and baseline eGFR, **c** percentage of patients in terms of % change from baseline in eGFR, **d** event rates and % change from baseline in eGFR. *eGFR* estimated glomerular filtration rate, *ESRD* end stage renal disease

Demographic and baseline clinical characteristics of the pooled ITT population from the EPPIC trials are shown in Table 1 and were similar between the AST-120 and placebo treatment groups. The distribution of baseline eGFR classified as <15, 15–20, 20–25, 25–30 and >30(mL/min/1.73 m^2^), is shown in Fig. 1a. Event rate percent of achieving both the primary endpoint and ESRD were greater in the patients with lower baseline eGFR (Fig. 1b).

**Table 1:** Demographic and baseline clinical characteristics of the pooled ITT EPPIC population

	AST-120, *N* = 1000	Placebo, *N* = 999	*P* value
Age, years, mean ± SD	55.4 ± 15.3	55.6 ± 14.8	0.74
Sex (%)			
Male	58.2	60.3	0.34
Race (%)			
White	80.7	78.6	0.57
Black or African American	7.3	8.9	
Asian	4.0	4.2	
Other	8.0	8.3	
CKD etiology (%)			
Diabetic nephropathy	40.4	40.4	0.98
Nondiabetic nephropathy	59.6	59.6	
Glomerulonephritis	25.2	28.9	
Nephrosclerosis	16.8	16.4	
Other	17.6	14.2	
Use of ACEI or ARB (%)			
Yes	84.4	84.2	0.89
Baseline sCr, mg/dL, mean ± SD	3.07 ± 0.88	3.14 ± 0.87	0.08
Baseline eGFR, mL/min/1.73 m^2^, mean ± SD	22.66 ± 7.93	22.04 ± 7.23	0.06
Baseline eGFR, mL/min/1.73 m^2^ (%)			
≤15	17.4	16.9	0.18
15–20	25.4	27.2	
20–25	23.3	24.3	
25–30	15.4	17.7	
>30	18.5	13.8	
CKD stage (%)			
Stage 3a	0.8	0.3	0.07
Stage 3b	17.7	13.5	
Stage 4	64.1	69.3	
Stage 5	17.4	16.9	
Baseline UP/UCr ratio			
*N*	998	995	
Mean ± SD	1.97 ± 1.33	2.01 ± 1.34	0.52
Baseline anemia status (%)			
Yes	70.3	71.1	0.66
BMI (kg/m^2^)			
*N*	998	999	
Mean ± SD	29.1 ± 6.4	29.1 ± 7.2	0.95

Race was self-reported

To convert sCr from mg/dL to mol/L, multiply by 88.4

Anemia was defined as a hemoglobin level <13.5 g/dL (men) or <12.0 g/dL (women)

Body mass index is the weight in kilograms divided by the square of the height in meters

*N* number of patients in the respective population, *SD* standard deviation, *ACEI* angiotensin-converting enzyme inhibitor, *ARB* angiotensin-II receptor blocker, *UP/UCr* urinary protein to urinary creatinine ratio, *BMI* body mass index

The distribution of the rate of change (%) in eGFR, classified as <−60, −60 to −40, −40 to −20, −20 to 0 and >0, is shown in Fig. 1c. Most patients experienced eGFR change between −20 and 0%, however, patients in this category had a similar low event rate compared to the population with a change in eGFR >0% (Fig. 1d). Higher event rates were observed in patients with higher percentage of change in eGFR (Fig. 1d).

The results of univariable and multivariable analyses of factors associated with the primary endpoint (triple composite of dialysis initiation, kidney transplantation, or doubling of the sCr level) and the secondary endpoint (change from baseline in eGFR %) are shown in Tables 2 and 3, respectively. In the multivariable analysis, sex, UP/UCr, anemia, use of ACEI/ARB, hematuria and systolic blood pressure (SBP), region and baseline sCr level were associated with the primary endpoint (Table 2), and UP/UCr, hematuria and region were associated with eGFR % change (Table 3). We therefore calculated the eGFR decline in the patients with hematuria positive and patients with a baseline UP/UCr ≥1.0. Since region was the stratified factor, analysis by regions was not conducted. eGFR decline in the hematuria-positive placebo-treated patients (*N* = 343, −7.01 mL/min/1.73 m^2^/year) was more rapid than in the hematuria-negative placebo-treated patients (*N* = 655, −3.89 mL/min/1.73 m^2^/year). Also, eGFR decline in placebo-treated patients with a baseline UP/UCr ≥1.0 (*N* = 706, −6.22 mL/min/1.73 m^2^/year) was more rapid than in placebo-treated patients with a baseline UP/UCr <1.0 (*N* = 289, −1.85 mL/min/1.73 m^2^/year). These results showed that the common covariates for the primary and secondary endpoints, UP/UCr ≥1.0 and hematuria, were independent risk factors for both ESRD occurrence and rapid disease progression in EPPIC trials.

**Table 2:** Univariable and multivariable stratified Cox analysis for primary endpoint

Baseline parameters	*N*	*n*	Univariable analysis		Multivariable analysis		*P* for interaction
			HR (95% CI)	*P* value	HR (95% CI)	*P* value	
Treatment							
Placebo	999	360	1.00		1.00		
AST-120	1000	350	0.92 (0.80–1.07)	0.32	0.89 (0.77–1.04)	0.15	–
Age, years mean ± SD, 55.5 ± 15.0							
≤65	1423	517	1.00		1.00		
>65	576	193	0.98 (0.83–1.15)	0.82	1.02 (0.86–1.22)	0.77	0.68
Sex							
Female	815	277	1.00		1.00		
Male	1184	433	1.08 (0.93–1.26)	0.27	0.83 (0.71–0.97)	0.02	0.28
Race							
White	1592	561	1.00		1.00		
Others	407	149	1.12 (0.93–1.34)	0.21	0.84 (0.68–1.03)	0.10	0.61
UP/Ucr mean ± SD, 1.99 ± 1.33							
<1.0	572	106	1.00		1.00		
≥1.0	1421	603	2.97 (2.41–3.65)	<0.01	2.62 (2.12–3.24)	<0.01	0.12
Anemia							
Positive	1402	580	1.00		1.00		
Negative	581	124	0.41 (0.34–0.50)	<0.01	0.57 (0.46–0.70)	<0.01	0.36
Use of ACEI/ARB							
Yes	1685	581	1.00		1.00		
No	314	129	1.39 (1.15–1.68)	<0.01	1.27 (1.05–1.55)	0.01	0.67
Hematuria							
Positive	700	309	1.00		1.00		
Negative	1298	400	0.59 (0.51–0.68)	<0.01	0.60 (0.52–0.71)	<0.01	0.91
SBP mean ± SD, 133.8 ± 13.9							
≤130	929	291	1.00		1.00		
>130	1070	419	1.37 (1.18–1.60)	<0.01	1.27 (1.08–1.48)	<0.01	0.06
BMI mean ± SD, 29.1 ± 6.8							
<25	580	218	1.00		1.00		
≥25	1417	490	0.93 (0.79–1.09)	0.37	0.88 (0.75–1.05)	0.17	0.07
Region							
North America	701	290	1.00		1.00		
Central/Latin America	428	137	0.82 (0.66–1.00)	0.05	0.87 (0.70–1.08)	0.21	0.89
Europe	870	283	0.74 (0.62–0.87)	<0.01	0.63 (0.51–0.77)	<0.01	0.61
Baseline sCr level							
≤3.0 mg/dL	1034	234	1.00		1.00		
>3.0 mg/dL	965	476	2.79 (2.39–3.27)	<0.01	2.52 (2.14–2.98)	<0.01	0.14
Diabetic nephropathy status							
No	1191	398	1.00		1.00		
Yes	808	312	1.25 (1.08–1.45)	<0.01	1.10 (0.93–1.31)	0.24	0.49

Race was self-reported

North America, Canada and United States of America; Central/Latin America, Argentina, Brazil and Mexico; Europe, Czech Republic, Germany, Spain, France, Italy, Poland, Russia and Ukraine

*N* number of patients in the respective population, *n* number of patients who had primary endpoint achievement, *SD* standard deviation, *HR* hazard ratio, *CI* confidence interval, *UP/UCr* urinary protein to urinary creatinine ratio, *ACEI* angiotensin-converting enzyme inhibitor, *ARB* angiotensin-II receptor blocker, *SBP* systolic blood pressure, *BMI* body mass index

**Table 3:** Univariable and multivariable mixed model analysis for change from baseline in eGFR % for secondary endpoint

Baseline parameters	*N*	Univariable analysis		Multivariable analysis		*P* for interaction
		LSM (95% CI)	*P* value	LSM (95% CI)	*P* value	
Treatment						
Placebo	976					
AST-120	980	2.12 (−1.08 to 5.33)	0.19	2.42 (−0.73 to 5.57)	0.13	–
Age, years mean ± SD, 54.2 ± 14.5						
≤65	1400					
>65	556	6.58 (3.03 to 10.13)	<0.01	2.97 (−0.72 to 6.68)	0.11	0.25
Sex						
Female	796					
Male	1160	0.57 (−2.69 to 3.84)	0.73	0.20 (−3.08 to 3.50)	0.90	0.75
Race						
White	1564					
Others	392	−0.47 (−4.49 to 3.53)	0.81	−2.05 (−6.49 to 2.38)	0.36	0.34
UP/Ucr mean ± SD, 1.71 ± 1.20						
<1.0	559					
≥1.0	1391	−15.74 (−19.23 to −12.26)	<0.01	−14.42 (−18.03 to −10.81)	<0.01	0.88
Anemia						
Positive	1371					
Negative	569	2.65 (−0.88 to 6.20)	0.14	2.06 (−1.61 to 5.73)	0.27	0.73
Use of ACEI/ARB						
Yes	1648					
No	308	3.04 (−1.36 to 7.44)	0.17	2.61 (−1.76 to 6.99)	0.24	0.01
Hematuria						
Positive	687					
Negative	1268	10.38 (7.04 to 13.71)	<0.01	7.16 (3.68 to 10.65)	<0.01	0.63
SBP mean ± SD, 132.9 ± 14.0						
≤130	912					
>130	1044	−2.80 (−6.01 to 0.41)	0.08	−1.35 (−4.60 to 1.89)	0.41	0.15
BMI mean ± SD, 28.8 ± 6.4						
<25	569					
≥25	1385	1.70 (−1.83 to 5.24)	0.34	0.68 (−2.92 to 4.30)	0.70	0.08
Region						
North America	679					
Central/Latin America	419	−5.76 (−10.17 to −1.36)	0.01	−6.06 (−10.48 to −1.63)	<0.01	
Europe	858	−6.22 (−9.86 to −2.58)	<0.01	−4.99 (−9.33 to −0.65)	0.02	0.22
Baseline sCr level						
≤3.0 mg/dL	1010					
>3.0 mg/d	946	−2.66 (−5.88 to 0.54)	0.10	−0.47 (−3.80 to 2.84)	0.77	0.12
Diabetic nephropathy status						
No	1171					
Yes	785	1.17 (−2.10 to 4.44)	0.48	0.73 (−2.83 to 4.30)	0.68	0.09

Race was self-reported

North America, Canada and United States of America; Central/Latin America, Argentina, Brazil and Mexico; Europe, Czech Republic, Germany, Spain, France, Italy, Poland, Russia and Ukraine

*N* number of patients in the respective population, *SD* standard deviation, *LSM* least squares means, *CI* confidence interval, *UP/UCr* urinary protein to urinary creatinine ratio, *ACEI* angiotensin-converting enzyme inhibitor, *ARB* angiotensin-II receptor blocker, *SBP* systolic blood pressure, *BMI* body mass index

The interaction test between AST-120 and clinically relevant factors showed a significant effect only for use of ACEI/ARB (*P* for interaction 0.01) on the eGFR % change. This result suggests that use of ACEI/ARB might have affected the observed effect of AST-120 in the overall trials.

**Fig. 2:**
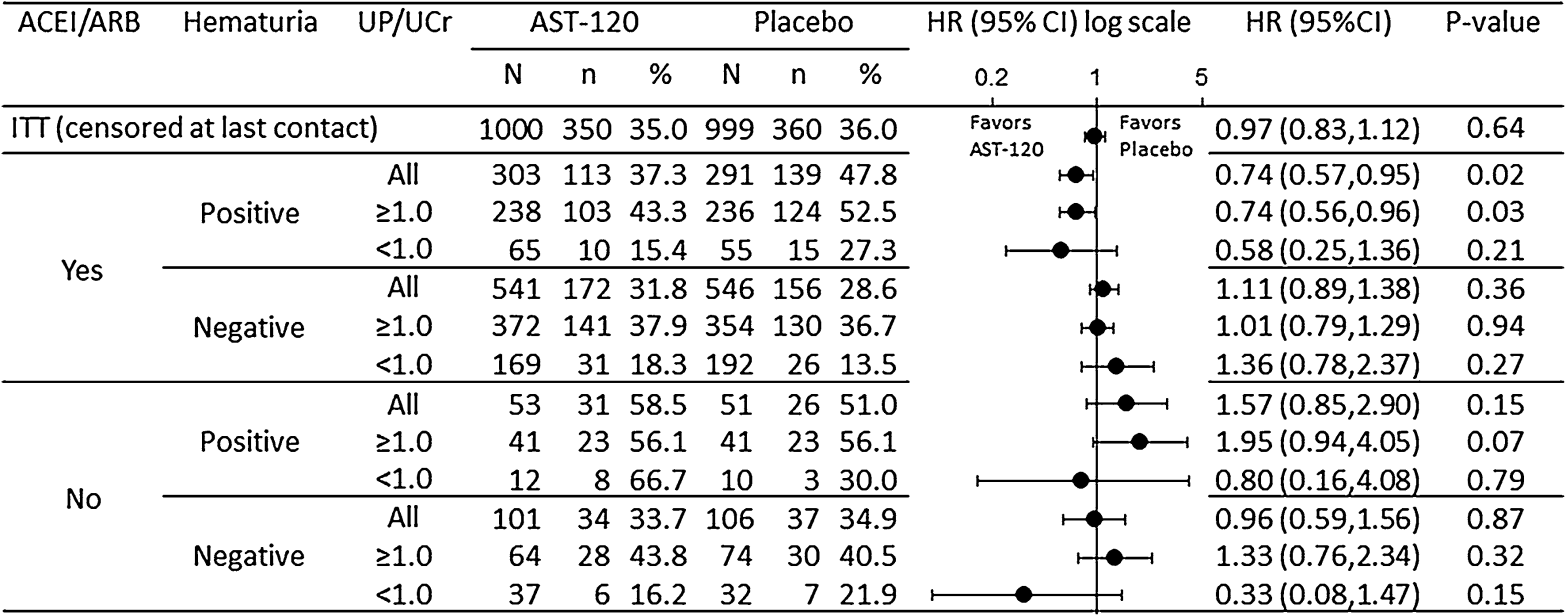
Effect of AST-120 on endpoint achievement. *N* number of patients in the respective population, *n* number of patients who had primary endpoint achievement, *ACEI/ARB* angiotensin-converting enzyme inhibitor/angiotensin receptor blocker, *CI* confidence interval, *HR* hazard ratio, *UP/UCr* urinary protein to urinary creatinine ratio. Patients without UP/UCr or hematuria data were excluded from the subgroup analysis

The effects of AST-120 on the primary endpoint in the subgroups with factors predicting rapid disease progression, UP/UCr and hematuria, and taking ACEI/ARB are presented in Fig. 2. In the current study, for the hematuria-positive group taking ACEI/ARB medication at baseline, additional treatment with AST-120 reduced the risk of achieving the primary endpoint (HR 0.74, 95% CI 0.57–0.95) (Fig. 2). A similar reduction was observed in patients with extremely rapid disease progression, who were hematuria positive with UP/UCr ≥1.0 and taking ACEI/ARB medication at baseline (HR 0.74, 95% CI 0.56–0.96). No differences were observed between the treatment groups in the hematuria-negative group or baseline UP/UCr <1.0 group, regardless of ACEI/ARB use.

**Fig. 3:**
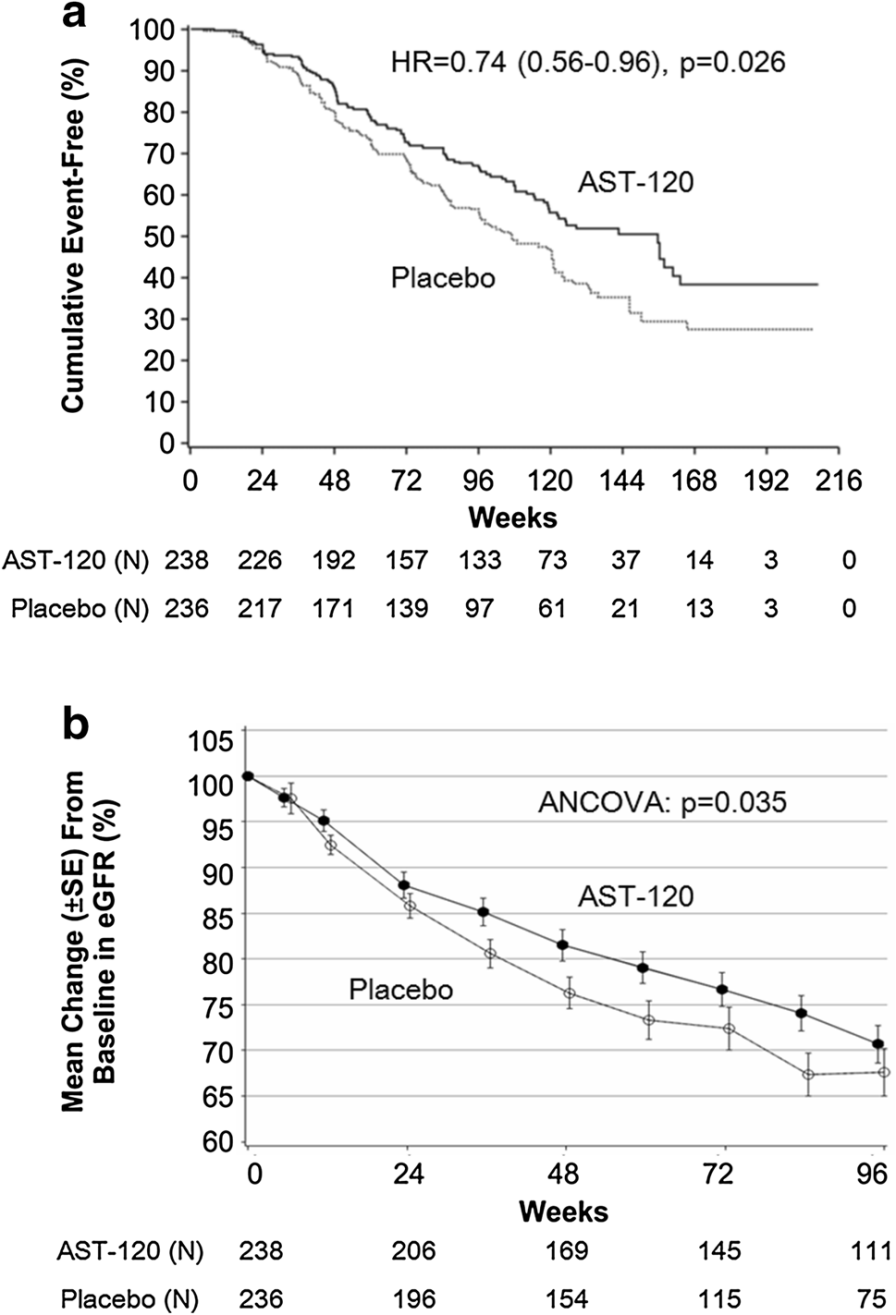
Kaplan–Meier analysis (**a**) and eGFR relative change from baseline (**b**) (UP/UCr ≥1.0, hematuria positive and baseline ACEI/ARB use). **a** Stratified Cox analysis, stratified factors: region, DN/non-DN, baseline sCr (3 mg/dL above or below). *N* number of patients in the respective population, *HR* hazard ratio. **b** Mixed-effects model was applied (ANCOVA). *ACEI/ARB* angiotensin-converting enzyme inhibitor/angiotensin receptor blocker, *eGFR* estimated glomerular filtration rate, *UP/UCr* urinary protein to urinary creatinine ratio

A K–M plot of event-free occurrence for the baseline hematuria positive and UP/UCr ≥1.0 with baseline ACEI/ARB use group is presented in Fig. 3a. In this high risk patient population, there was a lower primary endpoint occurrence the AST-120 treated group compared to placebo (*P* = 0.026). The declines in eGFR from baseline in the AST-120 group were smaller than the declines in the placebo group as shown in Fig. 3b (*P* = 0.035).

## Discussion

This post hoc analysis of the EPPIC study population demonstrated no correlation between CKD disease severity and disease progression, although both of them are the risk factors for the primary endpoint achievement. While the EPPIC study was successful in terms of enrolling patients with moderate-to-severe CKD and in terms of enrolling patients with UP/UCr ratios of ≥0.5, which was assumed to include patients with progressive disease, examination of the pooled placebo group revealed that the majority of the recruited patients had an eGFR change only between 0 and 20%. These patients were found to have a low occurrence of the primary composite endpoint and the ESRD endpoint as well as a low likelihood of achieving either of these endpoints.

Sample size determination for the EPPIC trials was based on the treated cohort of the Reduction in ENdpoints with the Angiotensin Antagonist Losartan (RENAAL) trial [8] in conjunction with further adjustment for differences in disease severity and heterogeneity within the RENAAL population. A median time to cumulative 50% event-free probability of approximately 124 weeks in the placebo group was estimated. However, the median time to event of the pooled placebo ITT population from the EPPIC trials was 180.1 weeks. In retrospect, the UP/UCr ≥0.5 inclusion criterion could be regarded as insufficient to include patients with rapid CKD progression.

To investigate the EPPIC population in detail, multivariable analyses were performed, and the results confirmed the well-known and established factors of baseline UP/UCr and hematuria, having a positive association between CKD progression and ESRD. While it is well established that the UP/UCr is highly associated with CKD prognosis, hematuria is recognized as a risk factor for disease progression in Japan [9, 10]. Recent Japanese CKD guidelines [4] state that both proteinuria and hematuria are associated with a high risk for CKD progression. A large-scale cohort study in Israel of young adults with hematuria demonstrated that persistent asymptomatic microscopic hematuria was a risk factor for ESRD onset [11]. A study of patients with stage 3–5 nondiabetic CKD demonstrated that microscopic hematuria was significantly associated with increased risk of ESRD, rapid renal function progression, and all-cause mortality, particularly in those with mild proteinuria [12]. Another study demonstrated that patients with advanced CKD with hematuria progressed significantly faster to ESRD as compared with patients with proteinuria alone [13]. Results from these studies propose that hematuria is a prognostic factor for renal outcomes in patients with CKD. There may be two reasons for the diminished importance of hematuria in clinical practice: its relationship to ESRD being less clear than the relation between ESRD and proteinuria, and the possibility of involvement of IgA nephropathy and thin basement membrane disease as the cause of the hematuria. In the EPPIC trials, 26% of patients with diabetic nephropathy had hematuria at baseline (data not shown); in another report, hematuria was observed in 30% of patients with diabetic nephropathy [14], which suggests that the occurrence of hematuria may not be limited to a particular disease. Results from this study suggest that close attention to hematuria in predialysis patients is warranted.

Multivariable analysis of the primary endpoint showed that high SBP and nonuse of ACEI/ARB were risk factors for ESRD (Table 2). The current standard of care for CKD includes blood pressure control. Medications that provide renin–angiotensin system blockade are the first-line choice for their possible renoprotective effect in reducing proteinuria and their strong hypotensive effect. In the EPPIC trials, AST-120 or placebo was added to standard therapy in adults with CKD; approximately 20% of patients were not taking ACEI/ARB medication at baseline. If patients not taking ACEI/ARB medication were excluded and the remaining patient data for progressing patients were analyzed to evaluate the add-on effects of AST-120 in standard therapy, there was a significant difference between treatment groups. There was no difference, however, between treatment groups in non-progressing patients taking ACEI/ARB. These results suggest that control of blood pressure and its treatment affected the occurrence of ESRD in this study. In addition, AST-120 may slow down disease progression in patients with rapidly progressing CKD taking standard of care medications such as ACEI/ARB. ACEI/ARB and AST-120 may have different modes of action, and may work in a mutually complementary manner in CKD treatment.

Subgroup analysis showed that AST-120 may delay the CKD progression in patients with rapid eGFR decline, hematuria and UP/UCr ≥1.0. Similar results were observed in studies in the Carbonaceous oral Adsorbent’s effects on Progression of chronic Kidney Disease (CAP-KD) study [15, 16] and in the Kremezin Study against Renal Disease Progression in Korea (K-STAR) [17, 18] which included patients with CKD progression based on observed pretreatment measurement. Results from both studies showed that AST-120 slowed eGFR lowering. It is probable that hematuria and UP/UCr could induce glomerular damage by increasing oxidative stress and inflammation which in turn could accelerate renal dysfunction. AST-120 may protect renal endothelial homeostasis by reducing the concentrations of uremic toxins such as IS, which induces inflammation [19].

In conclusion, higher UP/UCr levels and the prevalence of hematuria were shown to be risk factors for CKD progression in these post hoc analyses of the EPPIC study population. Furthermore, treatment with AST-120 was suggested to delay time to dialysis and prevent depression of renal function in the subgroup of patients in the EPPIC trials with elevated baseline UP/UCr and positive hematuria taking ACEI/ARBs. Since the major limitation of this analysis is its post hoc nature, further prospective studies in patients with the CKD progression risk factors of elevated UP/UCr, and hematuria are needed to confirm the results observed in these analyses, including the effects of AST-120.

## Electronic supplementary material

Below is the link to the electronic supplementary material.

Supplementary material 1 (PDF 275 kb)
