# Supplementary material for: Risk factors for progression of chronic kidney disease in the EPPIC trials and the effect of AST-120
Source: Clin Exp Nephrol. 2017 Jul 24;22(2):299–308. doi: 10.1007/s10157-017-1447-0 (PMC5838144; doi:10.1007/s10157-017-1447-0)
Supplement: Supplementary file 1 — Supplementary material 1 (PDF 275 kb) [file 10157_2017_1447_MOESM1_ESM.pdf]

Supplement 1

Study design

The EPPIC trials were conducted between July 2007 and February 2012 at 239 international sites in 13 countries (Canada, United States of America, Argentina, Brazil, Mexico, Czech Republic, Germany, Spain, France, Italy, Poland, Russia, Ukraine) to compare the effects of AST-120 with those of placebo with regard to renal outcomes in patients with moderate-to-severe CKD receiving standard therapy. Patients were randomly assigned 1:1 to receive treatment with 9 g/day AST-120 or placebo. The randomization stratification was according to the origin of kidney disease – non-diabetic or diabetic nephropathy, center (except outside the USA, where patients were stratified by country), and sCr of ≤ 3.0 mg/dL or > 3.0 mg/dL using screening values. AST-120 (administered as ten 300 mg capsules three times daily) or placebo was administered with meals and at least 1 hour after concomitant medication. Phosphate binders could be administered simultaneously with the study medication. EPPIC trials were event-driven studies, consisting of a 2-week prerandomization screening period, followed by a treatment period lasting until accrual of 291 primary renal end point outcomes in each study. The studies were anticipated to take a total of approximately 42 months to complete (24 months for enrollment and 18 months for treatment). However, the study duration exceeded 42 months for some patients to allow for the achievement of at least 291 primary endpoints.

Patients

Eligible patients were aged 18 years or older with moderate-to-severe CKD (defined as sCr level of 2.0–5.0 mg/dL [in male patients] or 1.5–5.0 mg/dL [in female patients] at screening) who were not expected to require dialysis or renal transplantation within 6 months of trial entry and who were expected to survive for 1 year or greater. All patients were required to demonstrate proteinuria or progressive deterioration in renal function based on either UP/UCr≥0.5 at screening or an increase in sCr level by >10% at the second evaluation conducted 3 months after the screening. Patients with hypertension must have had stable blood pressure in the 3 months prior to screening. If a patient was receiving antihypertensive therapy, treatment must have been stable and must have included either angiotensin converting enzyme inhibitor/angiotensin receptor blocker (ACEI/ARB) unless contraindicated.

Exclusion criteria included uncontrolled hypertension, obstructive or reversible kidney disease,

nephrotic syndrome (UP/UCr>6.0), adult polycystic kidney disease, uncontrolled arrhythmia or severe cardiovascular disease, immunosuppressive therapy or accelerated or malignant hypertension.

Outcome

The primary endpoint for this analysis was occurrence of any component of the triple composite endpoint: initiation of dialysis, kidney transplantation, or doubling of sCr levels. ESRD was defined as initiation of dialysis or kidney transplantation.

eGFR was one of the secondary endpoints described in the statistical analysis plan and was evaluated in this report. eGFR was measured at baseline, week 6, and every 12 weeks during the treatment period and at the early termination/discontinuation. The following formula was used to estimate eGFR:

$$\text{eGFR}(\text{mL/min}/1.73\text{m}^2) = 186 \times (\text{sCr})^{-1.154} \times (\text{Age})^{-0.203} \times (0.742 \text{ if female}) \times (1.210 \text{ if African-American})$$

Change from baseline in eGFR (%) was calculated during the first 96 weeks of treatment to elucidate the degree of renal disease progression

Statistical analysis

A pooled population of both EPPIC trials was used for all analyses.

The univariable and multivariable analyses were applied using the demographic and the baseline clinical characteristic as covariates to find the risk factors for the primary and the secondary endpoints. The Cox proportional hazards regression model and the mixed-effects model were applied for the primary and the secondary endpoints, respectively.

Baseline anemia was defined as hemoglobin level of less than 13.5 g/dL for men and as hemoglobin level of less than 12.0 g/dL for women.

Baseline hematuria status was classified as negative, “trace”, +1, +2, and +3, and patients with “trace” levels were included in the hematuria-positive population in this analysis. There was no obvious difference in the occurrences of primary endpoint if patients with “trace” hematuria status were included in the negative or positive group (Supplement 2). Both female and male patients were included in the analyses, despite the possibility that hematuria reported in female patients was due to menstruation. It was

confirmed that there was no difference in the results between female and male patients (Supplement 3).

For *post hoc* subgroup analyses, the same statistical methods used for analysis of the primary efficacy endpoint in the EPPIC trials were applied. The primary composite endpoint was analyzed using stratified Cox proportional hazards regression model with 95% confidence interval (CI); Covariate adjustments were CKD etiology, baseline sCr level, and region. Interactions between AST-120 and clinically relevant factors were also assessed in multivariable analyses adding interaction terms to a regression model.

The Kaplan-Meier (K-M) method and stratified Cox regression analysis were applied to compare time to the onset of primary endpoint between AST-120 and placebo groups with the following stratification factors: region [North America (Canada and US), Central/Latin America (Argentina, Brazil and Mexico) or Europe (Czech Republic, Germany, Spain, France, Italy, Poland, Russia and Ukraine)], baseline sCr level (above/below 3.0 mg/dL), and diabetic nephropathy status (yes/no). The proportional hazard assumption was checked using log-log plots of survival function versus survival time. The secondary endpoint, change from baseline in eGFR (%) over the first 96 weeks of the study, was analyzed using the mixed-effects model for repeated measures and analysis of covariance (ANCOVA).

The effect of AST-120 in the subgroups with factors predicting rapid disease progression was then assessed. Since current CKD standard of care includes ACEI/ARB therapy the effect of AST-120 in patients taking ACEI/ARB at baseline was assessed in this analysis.

Supplement 2. Subgroup analysis by hematuria status using stratified Cox proportional hazards regression for primary endpoint in pooled ITT population.

Hematuria	AST-120		Placebo		AST-120 vs Placebo		
	N	n(%)	N	n(%)	HR	(95% CI)	P-value
All	1000	350 (35.0)	999	360 (36.0)	0.97	(0.83, 1.12)	0.64
negative	643	206 (32.0)	655	194 (29.6)	1.07	(0.88, 1.31)	0.49
≥ trace	357	144 (40.3)	343	165 (48.1)	0.80	(0.64, 1.01)	0.06
≥ +1	217	87 (40.1)	197	95 (48.2)	0.83	(0.61, 1.12)	0.21
≥ +2	84	36 (42.9)	81	39 (48.1)	0.76	(0.46, 1.25)	0.27
≥ +3	14	4 (28.6)	12	6 (50.0)	0.79	(0.17, 3.68)	0.76

N, number of patients in the respective population; n, number of patients who had primary endpoint achievement; HR, hazard ratio; CI, confidence interval.

Supplement 3

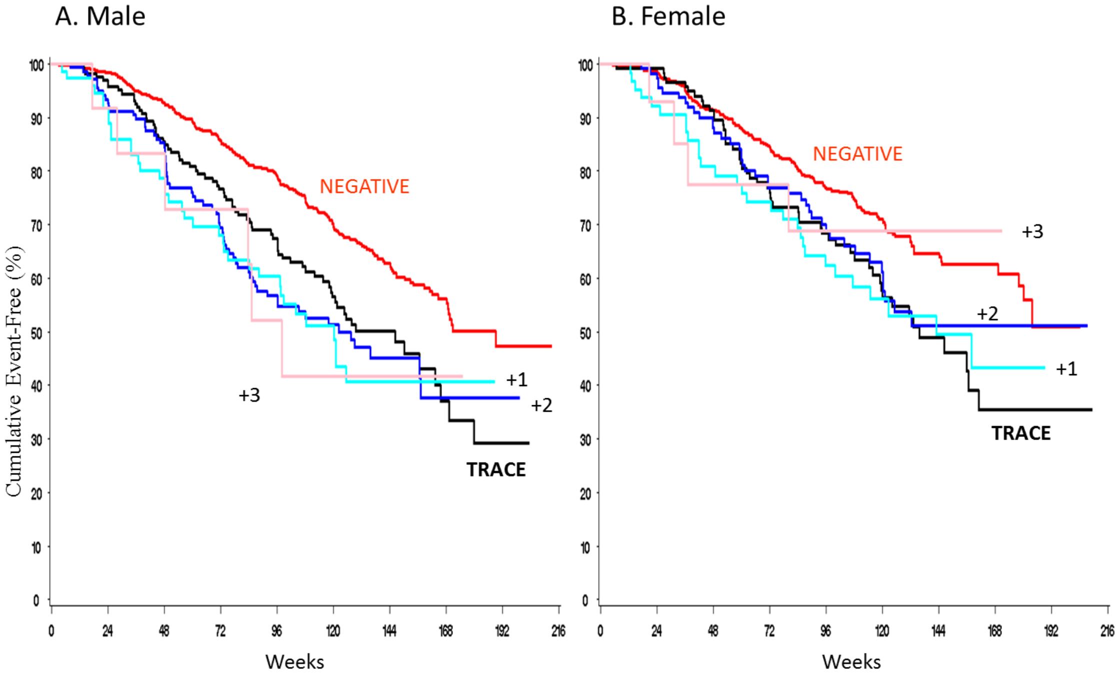

Kaplan-Meier analysis by the hematuria status on cumulative primary endpoint free rate in pooled placebo population.

A) Male, B)Female
